# Injuries and home advantage in the NFL

**DOI:** 10.1186/s40064-016-3432-6

**Published:** 2016-10-06

**Authors:** Marshall B. Jones

**Affiliations:** Professor and Chairman Emeritus, Department of Neural and Behavioral Sciences, College of Medicine, Pennsylvania State University, Hershey, PA USA

## Abstract

**Background:**

In the first decade of this century players in the National Football League, the NFL community, fans, even the public at large, became aware that multiple concussions, heretofore considered inconsequential, could have devastating consequences later in life.

**Results:**

Since 1978, each one of the 32 teams in the NFL plays 16 games in the regular season. In the 25 years from 1978 to 2004 home advantage in the regular season tended to increase with Game Number (1–16). Then in the following decade (2005–2014) it changed direction and tended clearly to decrease. The change in direction was highly reliable statistically.

**Discussion:**

The result reported in this paper is an association in time between two striking events, a new consciousness regarding the long-term consequences of concussions in football, and a change in the course of home advantage in the regular season. The paper then advances a possible explanation for this association. The home advantage may be equally well treated as an away disadvantage, the disadvantage being that away players tend to feel on the defensive, that both the hometown crowd and the officials are against them. Injuries put players on both teams on the defensive. The higher the percentage of players on a team who are injured or playing hurt (injury prevalence) the less likely it is that as-yet-uninjured players will adopt an attacking style of play. Injury prevalence increases linearly with Game Number. It turns out, however, that formal considerations require that injury prevalence be the same or close to it for teams playing at home and teams playing away. Therefore, the away disadvantage in total defensiveness (defensiveness due to playing away plus defensiveness due to injury) starts at 1 in the first game of the season, decreases steeply at first, and then decelerates as it approaches .5. This downward course of the away disadvantage in total defensiveness leads directly to a corresponding downward course of the home advantage in game outcome (by the teamwork theory of home advantage).

**Conclusions:**

Further research on the reported association or its explanation may be complicated by continued change in the association itself.

## Background

Home advantage is much the best established regularity in team sports. The five major professional sports for men in North America (baseball, basketball, football, ice hockey, and soccer) all play balanced home and away schedules;^1^ and in all five the home team wins more often than the away team, and not just in a few isolated studies but year after year for decades stretching back to the Second World War and earlier (Pollard and Pollard 2005; Jones 2015). In addition, the generality holds not just at elite levels of a sport but at less elite professional levels, amateur and college levels, internationally, and, where the sport is played by women, in both genders (Jones 2015). Home advantage is not just an interaction with circumstances, sometimes appearing and sometimes not, it characterizes team sports.^2^

This study began with an observation. In the playoffs of the National Football League (NFL) home advantage from 1995 to 2004 was 10 % points higher than it was from 2005 to 2014, 71 versus 61 %. The difference was not significant because the sample sizes were small, 100 in both decades. However, a 10-point percentage difference in home advantage is large, and it was suggestive. In July 2005 Dr. Bennet Omalu published his findings of chronic traumatic encephalopathy (CTE) in the brain of Mike Webster, a Hall of Fame center for the Pittsburgh Steelers. This article, published in the journal *Neurosurgery*, was the first scientific report of CTE in an NFL player. It was not, however, the first indication that NFL players had had that they were at risk for serious brain disorders. Six years earlier Steve Young, a Hall of Fame quarterback for the San Francisco 49ers, had been knocked unconscious for 30 min by a knee to the head and subsequently suffered post-concussive symptoms. Two years later Troy Aikman, another Hall of Fame quarterback, retired from the game citing concerns about concussions (he had had 10 of them) and back problems. In the 4 years after the Omalu report many players came forward and asked that their brains be donated for scientific study when they died. Some, including Terry Long and Junior Seau, committed suicide. The scientific reports were unanimous in indicating that repetitive blows to the head could lead to CTE. In 2009 the NFL, which had until then maintained an attitude of disdainful denial, gave way and issued guidelines that any player who showed symptoms of concussion should be sidelined for the rest of the game (Ezell 2015).

This history suggested a hypothesis. The year 2005 might not be the only year in which one could say that a new consciousness of the long-term consequences of injuries in football “began,” but it was close. Further, Binney (2015) had just published figures showing how new injuries and injury prevalence vary from 1 week to the next in the regular season. The incidence of new injuries remains flat from Week 2 to 17; but injury prevalence increases in a roughly linear way as the season wears on. The suggested hypothesis was that the original observation was somehow due to the general recognition that injuries in the NFL were serious and long-lasting. In addition, if injuries somehow brought about the drop in home advantage, then the increasing course of injury prevalence from week to week in the regular season should be accompanied by decreasing home advantage. The present paper is an exploration of this hypothesis.

## Methods

The data regarding home advantage were taken from Pro Football Reference (2015) on the internet. The data regarding injuries were taken from Injury Aftermath (Potter 2015), also on the internet. The inferential statistics used were familiar tests of significance, for correlations or proportions or differences between correlations or proportions.

This paper was reviewed and approved by the Ethics Committee of the Milton S. Hershey Medical Center and The Pennsylvania State University College of Medicine on May 12, 2016.

## Results

### Home advantage

**Fig. 1:**
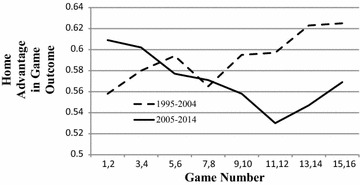
Home advantage in game outcome as a function of game number in two decades of the National Football League, 1995–2004 and 2005–2014

Figure 1 presents home advantage as a function of Game Number in two decades, 2005–2014 and 1995–2004. Home advantage is measured as the proportion of all games that are won by the home team, excluding ties.^3^ In both decades there were 32 teams and each one played 16 games in the regular season. There were therefore 16 games played each year at each Game Number, 16 rather than 32 because each game involved two teams. The entries reported in Fig. 1 are averages of 10 estimates of home advantage each one based on 32 data points. The Game Numbers were grouped in pairs to provide the estimates of home advantage with more reliability.

In the earlier decade, 1995–2004, home advantage increased more or less regularly (r = .226, *z* = 1.98, *p* < .05). In 2005–2014, the curve was less regular but clearly decreasing (r = −.28, *z* = 2.46, *p* < .02). The difference between the two correlations equaled .505 (*z* = 3.14, *p* < .001).^4^ Perhaps, however, the increasing curve in the earlier decade was not typical of the NFL considered more widely. Prior to 1978 each team in the NFL played fewer than 16 games in the regular season; and there were also 26 or fewer teams. Between 1978 and 1994, however, there were 15 seasons in which all teams played 16 games. Two seasons were abbreviated by strikes and were excluded. In the included 15 seasons the curve was also increasing, r = .14, but not significantly so. However, the difference between this correlation and the one for 2005–2014 equaled .42 (z = 2.86, p < .006). All told, therefore, home advantage for the 25 years preceding 2005 tended to increase with Game Number in the regular season. Then, in or around 2005, the curve changed direction and began to decrease with Game Number.

A further point should be mentioned. In Fig. 1 the curve for 1995–2004 appears to be roughly linear but the curve for 2005–2014 less so. In the first half of the season the curve for the more recent decade decreases quite regularly but then, in the second half of the season, it actually increases, primarily because of the mean value for games number 15 and 16. This value, however, may reflect an “end effect.” The primary issue at stake in the regular season is which teams qualify for the playoffs. By game 15 some teams have qualified and most have been mathematically excluded. Many teams rest their more valuable players in the last one or two games for fear of injury. If the value for games 15 and 16 is excluded, then the curve for the second half of the season is quite flat. In short, there is some reason to suppose that home advantage not only decreases with Game Number but does so in a decelerating manner.

### Injuries

The prevalence of injuries at home and away might explain these results. If injury prevalence in the decade from 2005 to 2014 increased more rapidly with Game Number at home than it did away, the decrease in home advantage would follow. It would only be necessary to suppose that injuries hurt a team’s chances of winning.

Fortunately, new injuries in the NFL are detailed each week during the regular season in a report by columnists at Football Outsiders entitled Injury Aftermath (Potter 2015). In each case the columnist notes the player, his injury, and his team. Whether the team was at home or away can then be determined from the schedule. New injuries are not, of course, the same as injury prevalence. The latter also depends on how long the injuries last, how many weeks an injured player is sidelined. Nevertheless, new injuries do provide some information relevant to the possibility that injuries were themselves distributed differently at home than away.^5^

The Injury Aftermath reports are available for all 16 games in 2014 and 2015 and for all games from 5 to 16 in 2013. In the first half of the season, Games 1–8, there were 194 new injuries at home and 186 away (all 3 years included). In the second half of the season, Games 9–16, the numbers were 197 at home and 212 away. The two proportions of home injuries, .51 and .48, are not statistically different. In addition, the difference goes the wrong way, toward relatively fewer injuries at home, not more, in the second half of the season.

## Discussion

To this point we have documented an association between the abrupt and unprecedented emergence of a new consciousness regarding the long-term consequences of football injuries, especially concussions, and an abrupt and unprecedented change in the course of home advantage. Still, what we have is an association, the coincidence in time of two striking events. It could be happenstance or there could be a connection, an explanation, a reason why the new consciousness has been accompanied by a change in the course of home advantage. The rest of this section advances such an explanation.

### Injury prevalence

Injury prevalence refers to “the percentage of players out with an injury or playing hurt” (Binney 2015). It is cumulative. A new injury can register only once, but that same injury can be included in the injury report for as many games as a player is at risk to play (not on injured reserve) but hurt or unable to play. For present purposes the key point is that the same injury which occurs at home is typically included in the injury report for the team when it plays away and vice versa. An injury which occurs at home in 1 week registers as an away injury if the player is still out and the team’s next game is away. The result is that injury prevalence at home and away converge rapidly to equality.

In a 16-game schedule there are 15 transitions from one game to the next, with 32 teams 480 transitions altogether. Many of these transitions are static, either home to home or away to away. The remainder involve a change in location, either home to away or away to home. In 2015 182 of the transitions or 38 % involved a change.

Every injury starts as a new injury. So suppose that in a given week 60 % of the new injuries affected teams playing at home and each injured player is out for a week. In the next week these same injured players and others not injured are distributed

$$\begin{aligned} .38 \times .6 & = .228\,{\text{moving}}\,{\text{to}}\,{\text{away,}}\,{\text{leaving}}\,.372\,{\text{still at home, and}} \\ .38 \times .4 & = .152\,{\text{moving}}\,{\text{to}}\,{\text{home,}}\,{\text{leaving}}\,.248\,{\text{still away}}. \\ \end{aligned}$$

The new injuries 1 week after they happen are distributed (.372 + .152=) .524 at home and (.228 + .248=) .476 away. If the players are still out a second week, the proportions at home and away drop to .504 and .496. The rate of convergence is steep and many players are injured for more than a week and some for much of the season.^6^

New injuries in the last 3 years (2013–2015) are distributed almost equally to players playing at home or away but, even if they weren’t, even if they were distributed as unequally as 60-40 or still more unequally, injury prevalence at home and away would still be approximately equal.

### The effects of injuries

The first effect of player injuries is obvious, namely, to reduce the effectiveness of a team’s player personnel. When a player is injured, he may be sidelined temporarily or placed on injured reserve. In the former case the team is simply short the services of one man for however long it takes him to return to action. In the latter case he is out for the season.^7^ The team may call up another player to replace him on the active roster but, at least on average, the replacement must be assumed to be not as good as the player who was injured, less experienced, less able, or not as current in training. In either case the team is weakened.

Players who are not injured may also be affected. How do they respond to a growing list of injured teammates? No studies have as yet been carried out. It is possible, however, to take a leaf from the economists’ playbook and ask what the rational response would be. Given the new consciousness regarding the long-term consequences of football injuries, especially concussions, wouldn’t the answer be “with more care to avoid injury either to himself or his teammates?” And wouldn’t that mean “with less willingness to attack and more attention to defense in the football sense of the word, that is, to keeping the opponents from scoring?” In short, wouldn’t it be, “more defensively?”

### Home advantage, defensiveness, and attention

The principal theories of home advantage are agreed that its cause has something to do with location, with the difference that one team is playing at home, in familiar circumstances, and a supportive hometown crowd while the other is playing away from home, in unfamiliar circumstances, and a crowd that is at best antagonistic and sometimes hostile; but there the agreement ends. The popular theory has it that the hometown crowd motivates the home and intimidates the away players as individuals. A second theory has it that the crowd effect is mediated not by the players but by the officials. Unconsciously for the most part, they accommodate the hometown crowd and give the benefit of the doubt or perhaps a little more to the home team (Balmer et al. 2001, 2003; Nevill et al. 2002).

The most recent theory (Jones 2015) treats the home advantage as an away disadvantage, namely, that the away players (in agreement with the first two theories) see the hometown crowd as hostile and the officials as accommodating. What makes this perception a disadvantage is that it puts the away players on the defensive. The hometown crowd is not necessarily hostile. Much of the time it is just rooting, but it never roots for the away team and some of the time it is hostile. To the away players the crowd’s attitude constitutes a threat, namely, that the home team will win and the crowd will rejoice.

The away disadvantage and football injuries are not, of course, the same thing. The outpourings of the hometown crowd are verbal, sometimes gestural, while football injuries are so many nonverbal facts. A threat, however, does not have to be verbal. A nonverbal thing may also be a threat if it is dangerous or likely to cause damage. Injury prevalence is such a thing. The injuries that happened to teammates may happen to players as yet unaffected, and they can’t help but know it. The hometown crowd and injuries to other players are both threatening. They both suggest circumspection, wariness, and a certain caution before committing oneself to action, both suggest defensive play.

The away disadvantage and injury prevalence are also alike in that what is threatened in both cases is delivered primarily by the opposing players. Players on the same side occasionally collide with injury to one or both. Most of the time, however, the collision is with an opposing player. Defeat is always at the hands of the other team. It makes sense, therefore, that a player pay close attention to the opposing players, the away players especially because they face a double threat, defeat by the home team with the enthusiastic approval of the hometown crowd as well as injury. One cannot, however, pay close attention to everything. The more one attends to the opposing players the less one can attend to one’s teammates.

Stress did not move to center stage in psychology until after World War II. When it did, one of the earliest and best-established results concerned the effects of stress on attention or “attentional narrowing” (Online Psychology Dictionary 2016; Combs and Taylor 1952; Driskell et al. 1999). Other investigators have called it by other names. For example, in an authoritative review of the literature, Easterbrook (1959) called it “a reduction in the range of cue utilization.” Under stress a person responds to a more limited range of cues than he or she would under other circumstances. If the stressor is a threat, the cues likely to produce a response become restricted to what is threatened and who or what is most likely to bring the threat about, at the expense of other cues which may be as or more relevant to the task at hand.

The threat posed to the away players by an antagonistic hometown crowd and the threat posed to players on both teams by a growing roster of injured teammates lead to a preponderant (though rational) concern with the opposing players rather than their teammates; and in both cases this concern is reinforced by the literature on attentional narrowing.

### Away disadvantage as a function of defensiveness due to injuries

American football is the quintessential team sport. For the offensive unit (the 11 players in possession of the ball) every play is planned beforehand. Each player has a job to do and each job is designed as part of an overall plan to advance the ball down the field. The execution of this plan depends entirely on timing. Each job must be done in precise temporal relation to what the rest of the team is doing. Teamwork is perhaps not quite as important to the defensive unit (the 11 players not in possession of the ball). The offensive players have only just learned which play will be run (when it was called by the quarterback) but the defensive players can only guess. Nevertheless, they also have roles to play and teamwork is key for them too. The backfield players (linebackers, corners, and safety) have not only to back each other up but also the defensive line. Even the linemen work in combination with each other, as, for example, when two or more work together to sack the opposing quarterback.

A focus on the opposing players is not just a distraction. It works systematically against teamwork. One cannot combine with teammates or back them up if one is not attending to them, if one is focused on an entirely different set of players. Teamwork therefore is degraded, both when a team plays away and when it experiences mounting injuries and the consequences of such injuries are well understood. The effect on home advantage when a team plays away is clear. The away team plays defensively, which involves an attentional focus on the home players which, in its turn, leads to poor teamwork; and good teamwork is necessary for quality team performance. The resulting away disadvantage is simply the double negative of home advantage.

In the decade from 2005 to 2014 injuries together with an understanding of their long-term implications affected both the home and away teams, injury prevalence was roughly equal, and both teams responded defensively. The effect is to reduce and ultimately to abolish the away disadvantage in total defensiveness and, except for occasional minor causes, the home advantage in games won or lost.

Away disadvantage in total defensiveness equals

$$\frac{{{\text{Defensiveness}}\, ( {\text{playing}}\,{\text{away)}} + {\text{Defensiveness}}\, ( {\text{away,}}\,{\text{injuries)}}}}{{{\text{Defensiveness}}\, ( {\text{playing}}\,{\text{away)}} + {\text{Defensiveness}}\, ( {\text{away,}}\,{\text{injuries)}} + {\text{Defensiveness}}\, ( {\text{home,}}\,{\text{injuries)}}}}.$$

In Game 1 there are as yet no injuries.^8^ What defensiveness there is is due to playing away. Hence, away disadvantage in total defensiveness equals

$$\frac{{{\text{Defensive}}{\kern 1pt} {\text{ness}}\, ( {\text{playing}}\;{\text{away)}}}}{{{\text{Defensive}}{\text{ness}}\, ( {\text{playing}}\;{\text{away)}}}} = 1.$$

Beginning, however, in Game 1 injuries pile up on both rosters, and equally. The result is that an equal amount of defensiveness, K, is added for both the away and home teams. The away disadvantage in total defensiveness then becomes

$$.5 < \frac{{{\text{Defensive}}{\kern 1pt} {\text{ness}}\, ( {\text{playing}}\;{\text{away)}} + K}}{{{\text{Defensive}}{\text{ness}}\, ( {\text{playing}}\;{\text{away)}} + 2K}} < 1.$$

**Fig. 2:**
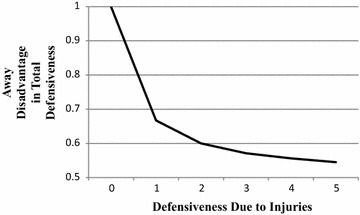
Away disadvantage in total defensiveness as a function of defensiveness due to injuries, where the latter is expressed in multiples of defensiveness due to playing away

As K increases the away disadvantage decreases until it eventually reaches .5 (no disadvantage). The decrease, moreover, is precipitous. K in Fig. 2 is given in multiples of the defensiveness due to playing away. When the defensiveness due to injury prevalence equals the defensiveness due to playing away, the away disadvantage in total defensiveness has already dropped two thirds of the way from 1 to .5.

The away disadvantage in defensiveness is, of course, not yet that in games won or lost. However, as Game Number increases, injury prevalence and defensiveness due to injuries also increase (linearly). Further, away disadvantage in total defensiveness, mediated by an away focus on opposing players and resultant poor teamwork, leads directly to away disadvantage in game outcome or, which is the same thing, home advantage in game outcome (Jones 2015). Thus the course of home advantage in games won or lost as a function of Game Number should be similar to the curve depicted in Fig. 2; and so it is (see the curve for 2005–2014 in Fig. 1). It is only necessary to suppose that by mid-season defensiveness due to injuries has exceeded defensiveness due to playing away.

## Conclusions

This paper has reported an association in time between the new consciousness that concussions in football may have devastating consequences later in life and a reversal in the course of home advantage in the regular season. It also advanced a theory to explain that association. Next tasks in research include a closer examination of the association itself and testing in new or additional ways of the theory offered here to explain it. Both of these tasks may be complicated by continued change in the association itself.

The present study was begun midway in the 2015 season. At this writing the season has been completed. In the first half of 2015 the home team won 74 and the away team 54 games for a home advantage of .58. In the second half of the same season, the home and away teams both won 64 games or no home advantage at all. Relative to the 10 preceding years, the mean of .58 ranks just below the median, while that in the second half is much lower than in any year of the preceding decade. In short, the process depicted in Fig. 1 is still continuing and deepening, and the possibility cannot be excluded that it will continue to do so for quite some time. Yet even so, even at this early stage in its development, the new consciousness about injuries in the NFL seems already to have altered a key feature of the sport.

## Footnotes

1 In football the schedule is not completely balanced. Within divisions it is; each team plays two games with every other team in the division, one at home and the other away. The rest of the schedule is balanced as to home or away but a team plays only one game with teams outside its division.

2 In individual sports the home advantage is small or nonexistent (Jones 2013, 2015).

3 Ties are possible in the NFL but rare, less than half a percent.

4 The correlations were run over all 80 estimates of home advantage (one for each year and each pair of Game Numbers).

5 It should also be noted that the information in Injury Aftermath does not appear to be complete. The text written by the columnist is followed by comments from readers. Occasionally, a reader mentions an injury not noted by the columnist. These comments are often themselves incomplete and were not included in the counts made by the author.

6 Injuries that occur in the last game of the regular season must, of course, remain as they are. However, injury prevalence in the last game is three times as large as new injuries (Binney 2015), and the two thirds carried over from earlier in the season will already have converged for at least one game and weigh heavily in favor of equality.

7 In 2012 the players’ union and the NFL reached an agreement that one player could be brought back from the injured reserve list.

8 In fact, there usually are some injuries prior to Game 1, in practice and the preseason, but these injuries have been discounted for the purposes of this discussion. Including them would simply reset the curve in Fig. 2 to begin somewhat below 1.
